# BEACON: automated tool for *B*acterial G*E*nome *A*nnotation *C*omparis*ON*

**DOI:** 10.1186/s12864-015-1826-4

**Published:** 2015-08-18

**Authors:** Manal Kalkatawi, Intikhab Alam, Vladimir B. Bajic

**Affiliations:** Computational Bioscience Research Centre (CBRC), King Abdullah University of Science and Technology (KAUST), 23955-6900 Thuwal, Kingdom of Saudi Arabia

**Keywords:** Genome annotation comparison, Gene function annotation, Functional annotation of bacterial genomes, Annotation methods, Bioinformatics

## Abstract

**Background:**

Genome annotation is one way of summarizing the existing knowledge about genomic characteristics of an organism. There has been an increased interest during the last several decades in computer-based structural and functional genome annotation. Many methods for this purpose have been developed for eukaryotes and prokaryotes. Our study focuses on comparison of functional annotations of prokaryotic genomes. To the best of our knowledge there is no fully automated system for detailed comparison of functional genome annotations generated by different annotation methods (AMs).

**Results:**

The presence of many AMs and development of new ones introduce needs to: a/ compare different annotations for a single genome, and b/ generate annotation by combining individual ones. To address these issues we developed an Automated Tool for *B*acterial G*E*nome *A*nnotation *C*omparis*ON* (BEACON) that benefits both AM developers and annotation analysers. BEACON provides detailed comparison of gene function annotations of prokaryotic genomes obtained by different AMs and generates extended annotations through combination of individual ones. For the illustration of BEACON’s utility, we provide a comparison analysis of multiple different annotations generated for four genomes and show on these examples that the extended annotation can increase the number of genes annotated by putative functions up to 27 %, while the number of genes without any function assignment is reduced.

**Conclusions:**

We developed BEACON, a fast tool for an automated and a systematic comparison of different annotations of single genomes. The extended annotation assigns putative functions to many genes with unknown functions. BEACON is available under GNU General Public License version 3.0 and is accessible at: http://www.cbrc.kaust.edu.sa/BEACON/.

**Electronic supplementary material:**

The online version of this article (doi:10.1186/s12864-015-1826-4) contains supplementary material, which is available to authorized users.

## Background

Genome annotation is used to identify and denote function of different segments in a genome sequence [1] and forms a basis for many downstream genome analyses. Several annotation methods (AMs) for eukaryotes [2] and prokaryotes [3] have been developed. This multitude of AMs brings some natural questions such as those regarding the strengths, weaknesses and differences of these AMs and annotations generated by them, as well as a possibility to combine them to extend annotations of individual AMs. In spite the fact that a tool with such utilities will be of great help to researchers in the field, the public automated systems that provide detailed comparison between annotations are not available. Here, we present a simple, yet effective, Automated Tool for *B*acterial G*E*nome *A*nnotation *C*omparis*ON* (BEACON), which addresses these issues for functional annotations of prokaryotic genomes.

BEACON is capable of generating rapid, comprehensive, visual and informative analytical results. It can be used to: 1) compare annotations from multiple AMs relative to a selected reference annotation (for example from the National Centre for Biotechnology Information (NCBI) [4] published records, or BROAD Institute [5]); 2) perform annotation comparison between annotations obtained by different AMs for the same genome; 3) generate extended annotation for gene function when different AMs assign different functions to the similar gene (this annotation we denote as the EA); 4) expand EA by adding uniquely annotated genes from other annotations (this annotation we denote as the EUA). For example, one of the potential uses of BEACON can be to update annotations for large number of microbial genomes that are deposited long time ago and are not updated since then [6]. In these cases, multiple AMs can be deployed by BEACON.

There are many AMs used to annotate prokaryotic genomes with different tools being developed. For example: the NCBI Prokaryotic Genome Automatic Annotation Pipeline (PGAAP) [7], the Automatic Annotation of Microbial Genomes (AAMG) [8], the Rapid Annotations using Subsystems Technology (RAST) [9], the BG7 system [10], the rapid prokaryotic genome annotation Prokka [11], the prokaryotic annotation pipeline from the Institute for Genome Science (IGS) [12], and the Integrated Microbial Genome Expert Review system (IMG/ER) [13]. The annotation output of these AMs for the same genome may vary considerably since there are no commonly accepted standards for gene function annotation process [14]. A few methods [15–18] were used to evaluate and compare genome annotations, each having its advantages and drawbacks. An annotation confidence score (ACS) scheme was introduced [15] to provide a score to the annotation of particular genomes based on a group of selected reference genomes. ACS method that is based on genome comparison approach may reduce the number of gene annotations that have to be checked manually. However, it depends on the number of reference genomes and their phylogenetic distances. Another method [16] was introduced for comparing different annotations after removing hypothetical genes (those with the undefined function). Nevertheless, this method is not automated. In [17], the *Halorhabdus utahensis* (*H. utahensis*) genome was used to compare three different functional genome annotations. The method involved some manual procedures as well. A semi-automatic protocol to compare functional genome annotations is proposed in [18]. The system from [18] is difficult to setup and test due to complex input requirements and the need for interpretation of the reported results that could be subjective. The method implemented in BEACON provides a fully automated, simple and quick comparison of genome annotations generated by multiple AMs and addresses the four points indicated previously. Its EA provides the extended annotation of gene function increasing the number of genes annotated with functions. This increases the number of genes with assigned function (in our experiments up to 27 %) as compared to individual annotations. On the other hand, EUA, in addition to EA, expands the gene set of individual annotation by adding uniquely annotated genes from other annotations.

In what follows we present the method used, the implementation of BEACON, the evaluation datasets, and the results obtained by applying BEACON to *H. utahensis* genome annotations generated by three different AMs. In Additional file 1: Section 3 we provide the comparison results of annotations by different AMs for the additional three genomes (*Escherichia coli (E. coli)* K-12 strain*, E. coli* TY2482 strain and *Candidatus Carsonella ruddii DC* (*C. ruddii DC*)).

## Implementation

### Beacon: method and implementation

**Fig. 1:**
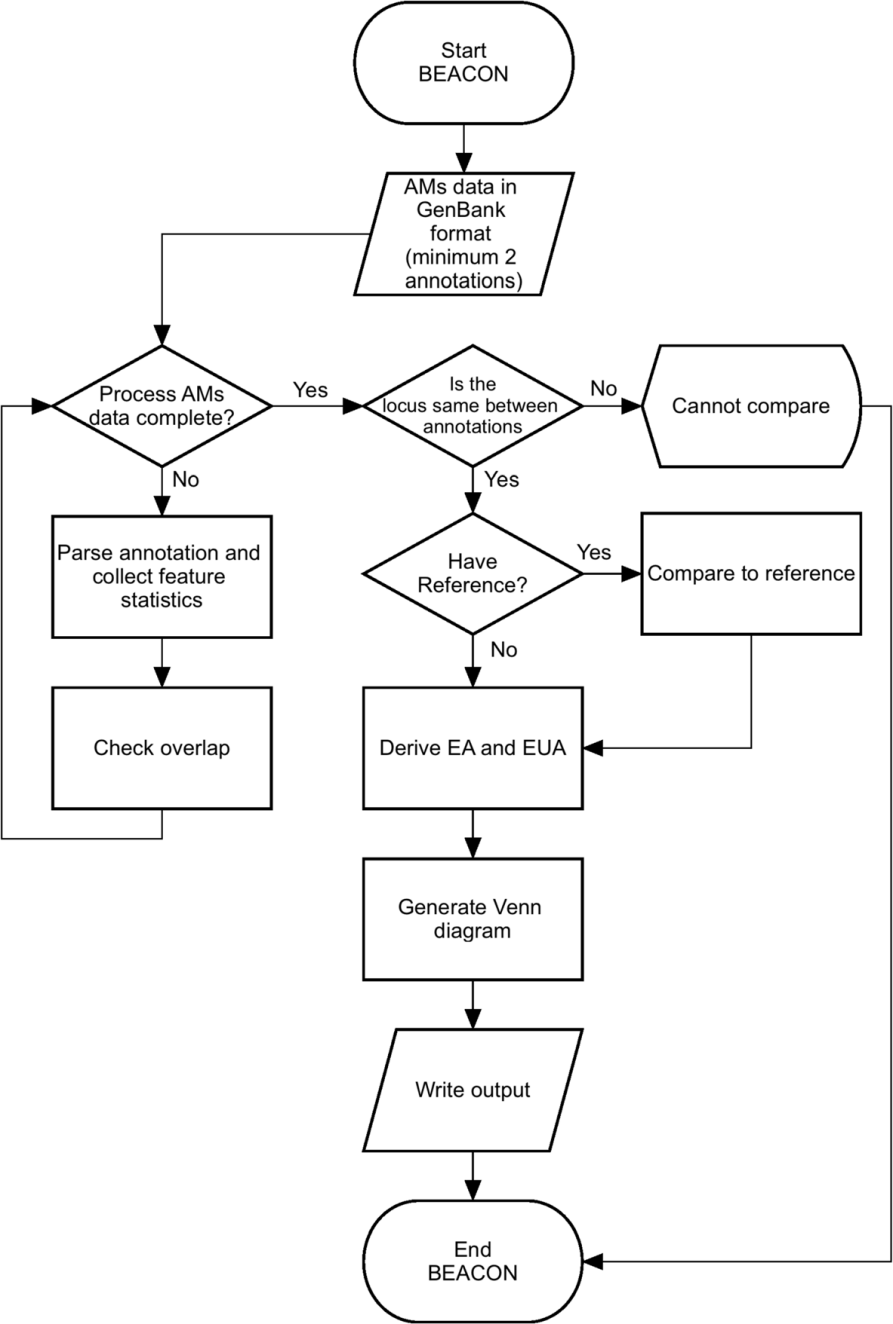
BEACON workflow. This diagram illustrates the flow of the processes in BEACON tool

In order to have automated, rapid, simple and informative genome annotation comparison we developed a tool, BEACON, which can analyze and compare annotations generated by multiple AMs and extend the annotation generated by an individual AM. BEACON is implemented in C++ and its workflow is outlined in Fig. 1.

#### Annotation comparison

The first functionality of BEACON is the comparison of multiple annotations and, as mentioned above, it works with or without a reference annotation. Initially, BEACON collects basic relevant statistics from each of the annotations. This information includes: the number of coding DNA sequences (CDSs), transfer RNA (tRNA), ribosomal RNA (rRNA), non-coding RNA (ncRNA), overlapping genes, pseudogenes, frameshifted genes, discontiguous genes, genes encoding hypothetical products (e.g. orphan genes), genes encoding non-hypothetical products (e.g. genes encoding proteins with known function), and whether they are with gene symbol, without gene symbol, or conserved [19] according to the existing annotations in public databases. Then, BEACON starts the annotation comparison process. For comparison to a reference annotation, let us assume that for a genome sequence we generated an annotation *X* using one AM and we want to compare this annotation to a reference annotation, *Z* (note that BEACON can accept several annotations for the comparison). BEACON first parses the *Z* annotation, records the gene count and detailed information about each gene, such as locus name, locus tag, gene strand, gene type: CDS, rRNA, tRNA, ncRNA, genomic start and stop positions of a gene, whether the gene is pseudogene or frameshifted based on its annotation, and the other gene information. BEACON then finds overlapping genes within the same annotation, either with significant or short overlap. By default, BEACON considers two genes on the same strand significantly overlapping if they contain common locations and if the start and stop positions of the shorter gene relative to that of the longer one are deviating for no more than a given offset (default is 2 %) of the length of the shorter gene. Greater deviations are considered by BEACON as a short overlap. However, users can define other percentage of mismatch of gene ends for this purpose. BEACON then parses annotation *X*, keeping track of gene count and information on individual genes to compare those to the reference annotation *Z*. In the process, BEACON compares the start and stop locations of each gene in annotation *X* (pair-wise comparison in case there are more than one input annotation) to the start and stop positions of all the genes in annotation *Z*. As a comparison condition, genes should be on the same locus, same strand and if one of the genes is pseudogene, we will consider identical or significantly overlapping genes from other AMs pseudogene as well. Additional comparison condition for CDS genes is to be in the same frame, then BEACON reports the same frame or frameshifted genes. BEACON then assigns a status to each gene in annotation *X*. These genes could be denoted as identical, similar, unique with overlap or unique without overlap:

- Genes from annotation *X* that completely overlap (exactly the same start and stop genomic locations) with genes in annotation *Z* will be identical genes for *X* and *Z*.
- Genes from annotation *X* that are not overlapping with any gene from annotation *Z* will be unique for *X* and vice versa, genes from annotation *Z* that are not overlapping with any gene from annotation *X* will be unique for *Z*.
- All other genes are overlapping to some extent:
  - ▪ If genes share some common locations and if the start and/or stop locations are deviating between the two annotations for no more than a given offset (e.g. 2 %) of the length of the shorter gene (significant overlap), and in the same frame for CDS genes, then such genes will be similar genes for *X* and *Z*.
  - ▪ If this mismatch is larger than the offset (short overlap), these genes are considered unique genes with overlap for *X* and *Z*.

At the end of the comparison, BEACON calculates a similarity score between *X* and *Z* annotations according to (1):

1 $$ SimilarityScore=\frac{Identical+ Similar}{Tota{l}_x+ Tota{l}_z}\ast 2\ast 100 $$

where *Identical* and *Similar* are the numbers of identical and similar genes, respectively, between annotations *X* and *Z. Total*_*x*_ and *Total*_*z*_ are the total number of genes in annotations *X* and *Z*, respectively. Note that this similarity score is symmetric. In other words, similarity score between *X* and *Z* relative to *X* or relative to *Z* will be the same.

In the case when no reference annotation is available, or if one wishes to mutually compare multiple annotations, BEACON processes each annotation as previously mentioned including overlap checking. After that, BEACON finds common genes based on their genomic location between input annotations with all possible combinations of these annotations. BEACON uses this information to generate Venn diagram based on the R [20] ‘VennDiagram’ package [21], that illustrates intersections between annotations. Venn diagram can be generated for up to five annotations, otherwise BEACON just reports results textually.

#### Deriving extended annotations

The second functionality of BEACON is the use of the annotations generated from the comparison stage to derive an EA of gene function and to expand the individual AM annotations. BEACON provides a list of common genes (those that significantly overlap, on the same locus, same strand, and in the same frame for CDS genes) between two or more annotations. The original annotations of gene function from different annotations are enlisted and combined to generate EA. Since comparing the text of gene description provided by different AMs is not straightforward, BEACON prefers to list all the descriptions from the multiple AMs and leave it to the user to choose one of the AMs or use a combination of them. In a separate list BEACON provides genes unique to each of the annotations together with their annotation of function. These are combined with EA and produce EUA. The EUA is also provided in a clean form where the pseudogenes and frameshifted ones are excluded. The extended annotation information is provided in both tabular and the gff file format, so it can be used further to visualize or to carry out other analysis using other software. Through this mechanism one may complement individual AM annotation of gene functions (see results from one example in Table 1), where the difference between original annotation and EA of genes, that might encode orphan or functional proteins, is presented. Both EA and EUA can benefit users to get more comprehensive information about the genome in question.

**Table 1:** Statistics for different annotations for *H. utahensis* genome along with the extended annotations. For orphan and functional genes we show the actual number of genes and the percentage relative to the total number of annotated genes

Annotation features	NCBI		AAMG		RAST		Extended annotations		
	Original	Complemented by annotation of function from AAMG and RAST	Original	Complemented by annotation of function from NCBI and RAST	Original	Complemented by annotation of function from NCBI and AAMG	EA	Unique	EUA
CDS	2998	2998	3040	3040	3041	3041	2980	698	3678
rRNA	4	4	3	3	3	3	4	0	4
tRNA	45	45	45	45	45	45	45	0	45
ncRNA	1	1	0	0	0	0	0	1	1
frameshift/Pseudo	0	0	0	0	0	0	0	0	0
Total	3048	3048	3088	3088	3089	3089	3029	699	3728
Orphan genes	1014 (33.27 %)	777 (25.49 %)	885 (28.66 %)	837 (27.10 %)	1203 (38.94 %)	819 (26.51 %)	672 (22.19 %)	399 (57.08 %)	1071 (28.73 %)
Functional genes	2034 (66.73 %)	2271 (74.51 %)	2203 (71.34 %)	2251 (72.90 %)	1886 (61.06 %)	2270 (73.49 %)	2357 (77.81 %)	300 (42.92 %)	2657 (71.27 %)

#### Usage

The BEACON tool is accessible at: http://www.cbrc.kaust.edu.sa/BEACON/. The source code is also available for download for the command line use at the BEACON website and in Additional file 2. For the command line use, the user has to specify output directory, short name for the genome, similarity offset, and files with the annotations in the GenBank format, as well as a label for each of these annotations. These annotations will be read by BEACON. BEACON will mutually compare all annotations (all against all). A user can specify the first listed genome annotation as a reference annotation. A detailed documentation for the usage of both web-interface and command-line is described in Additional file 1: Section 1 and is provided on the BEACON’s website also. BEACON is provided under GNU General Public License version 3.0.

### Data

In this study we considered annotations from three AMs (NCBI, AAMG and RAST) for four microbial genomes, namely *H. utahensis* [22]*, E. coli* K-12 strain*, E. coli* TY2482 strain and *C. ruddii DC* [23]*.* The *H. utahensis* genome annotations were obtained from NCBI, and also generated by RAST and AAMG. The *E. coli* K-12, *E. coli* TY2482 and *C. ruddii DC* data and annotations were obtained from [8] with additional annotations generated by RAST. The comparison of AMs for the last three genomes is provided in Additional file 1: Section 3. All of these data sets are available at the BEACON website.

## Results

As the first example to illustrate capabilities of BEACON, we used *H. utahensis* genome to compare the annotations by AAMG and RAST to the annotation from NCBI. A detailed study of *H. utahensis* genome was presented in [17]. *H. utahensis* genome annotations were generated by RAST and AAMG. The resultant GenBank files from RAST and AAMG were submitted to BEACON together with the NCBI annotation for *H. utahensis* as the reference. The comparison results were obtained in less than 7 s on a typical laptop (MacBook Pro, 2.7GHz, Intel Core i7).

In the other examples (Additional file 1: Section 3), the AAMG and RAST annotations were compared against the NCBI annotation for both *E. coli* K-12 and *C. ruddii DC*. For *E. coli* TY2482, AAMG, RAST and BG7 [10] annotations were compared against the BROAD annotation.

The BEACON output is categorized into five groups: ‘annotations information’, ‘comparison to reference’, ‘extended annotations (EA, EUA, EUA_Clean, unique)’, ‘Venn diagram’ and ‘Web’. The detailed description of the analytical tab-delimited output of each of these groups is found in Additional file 1: Section 2 and Figures S4-S16. The results are visualized using Google’s chart API [24] to display information in histograms that show distribution of statistical and comparison data (Fig. 2), and a Venn diagram that illustrates intersections between annotations (Fig. 3).

**Fig. 2:**
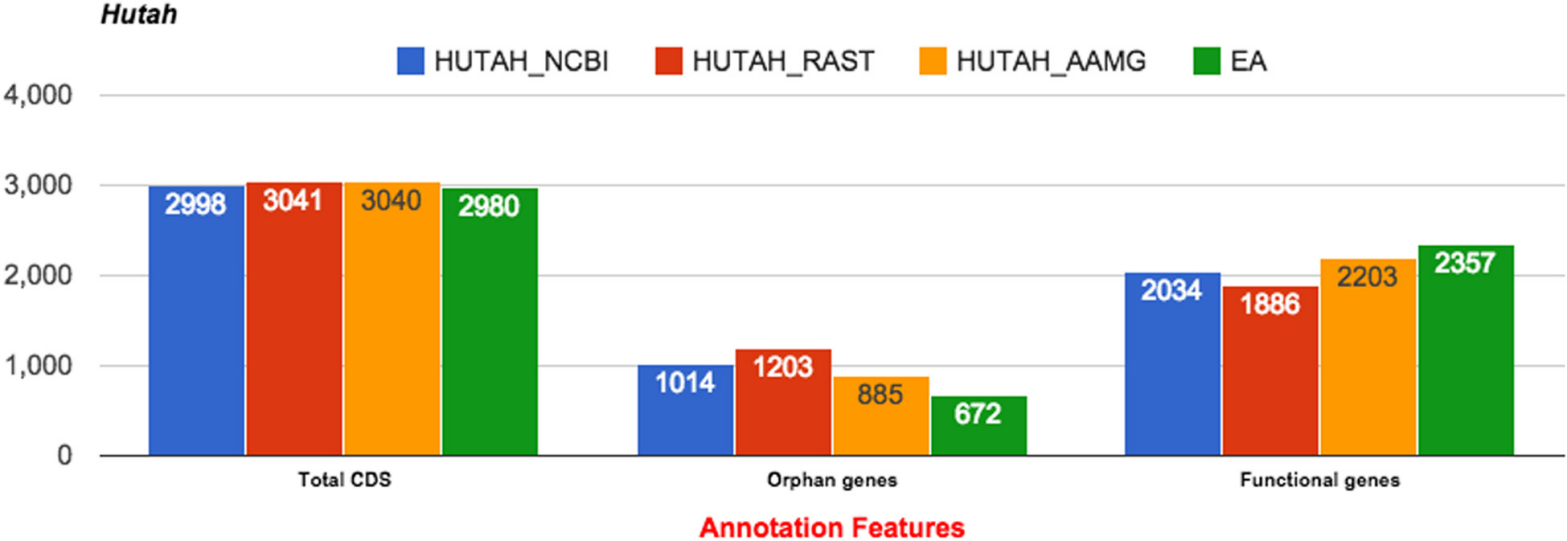
AMs features comparison stats. Histogram that shows the distribution of the statistical and comparison data of *H. utahensis* genome

**Fig. 3:**
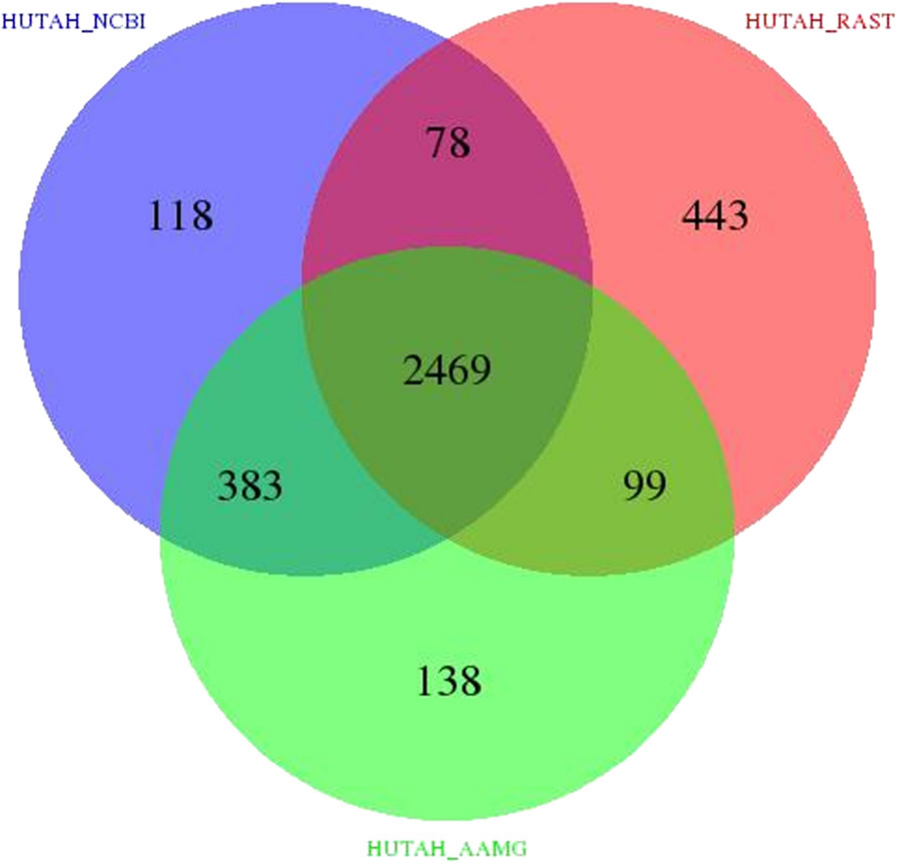
Venn diagram showing unique/common genes from different AMs. Venn diagram that illustrates intersections between different annotations of *H. utahensis* genome

The statistical comparison results of *H. utahensis* genome are summarized in Table 1 along with EA information (more detailed results are found in Additional file 1: Tables S1-S3). One can observe that EA among two or more AMs has the least percent of genes with undefined protein functions and the maximum percent of genes annotated as functional (difference is 16 % of absolute scale for the *H. utahensis* genome, but can be larger for other genomes as shown in Additional file 1: Section 3), as compared to annotations from NCBI, AAMG and RAST (difference is 27 % for *E. coli* TY2482). Therefore, in the case of the *H. utahensis* genome, EA is the ‘best’ overall. As the ‘best’ annotation we consider here the one that has the largest number of genes with annotated function and the least number of genes with hypothetical functions. We have to highlight that the functions assigned to genes by different AM could be putative. However, as such they still hint to potential gene functions. We do not assess the quality of annotations generated by individual AMs. Table 2 illustrates the result of annotations comparison, showing that the AAMG annotation is 92.94 % similar to the one from NCBI, whereas the RAST annotation is only 83.02 % similar. The results of other tested genomes are shown in Additional file 1: Tables S4-S12 and Figures S17-S19 that shows EA to be with the largest proportion of genes with annotation of function and the AAMG annotation appears to be most similar to the reference for all different genomes we analyzed.

**Table 2:** AAMG and RAST annotations compared to the NCBI annotation that is taken as the reference. False Negatives (FN) are genes that exist in the NCBI annotation but are not predicted by an AM. False Positives (FP) are genes predicted by an AM but not present in the NCBI annotation

Gene calls	Genes annotated by RAST	% of NCBI genes	Genes annotated by AAMG	% of NCBI genes
Detected identical	2421 (CDS = 2376 rRNA = 0 tRA*N =* 45 ncRA*N =* 0)	79.43 %	2780 (CDS = 2732 rRNA = 3 tRA*N =* 45 ncRA*N =* 0)	91.21 %
Detected similar	126 (CDS = 123 rRNA = 3 tRA*N =* 0 ncRA*N =* 0)	4.13 %	71 (CDS = 71 rRNA = 0 tRA*N =* 0 ncRA*N =* 0)	2.33 %
FN - Short overlap	74	2.43 %	32	1.05 %
FN - No overlap	427	14.01 %	165	5.41 %
FP - Short overlap	13	-	0	-
FP-No overlap	529	-	237	-
Similarity score		83.00 %		92.93 %

## Discussion

Our intention is to provide a tool (BEACON) that can help in analysing and comparing different annotations that could be generated by different AMs. We have illustrated the capabilities of BEACON by performing comparisons of several annotations for 4 genomes. While this is not a proof of the efficiency of BEACON, it is sufficient to point out its potential for such types of analyses.

Currently, BEACON is limited to the use of the GeneBank format annotation. In the case when no reference annotation is selected/provided, the comparison is made in all-against-all fashion. BEACON does not resolve inconsistencies of different annotations, but lists them and make them available to users to analyse and choose from. For this reason, BEACON, for example, does not correct the stop and start positions in a Genbank file. In future versions of BEACON we intend to address some of these issues.

## Conclusions

We developed a fast publicly accessible tool BEACON for an automated and a systematic comparison of different genome annotations that also generates extended annotations. It can help users decide which annotation suits better their studies, or if they will use a combination of the genome annotations. It also provides possibility to increase the number of genes to which a function could be assigned, thus complementing annotations by individual AMs and extended it by uniquely annotated genes from different annotations. We believe that BEACON may help improve the quality of bacterial genome annotations deposited in public databases when multiple annotations are available for the same bacterial genome.

## Availability and requirements

The BEACON web tool is accessible at the following address: http://www.cbrc.kaust.edu.sa/BEACON/.BEACON is free for use by academic and non-profit users. The input is GenBank-formatted annotation files. The source code (implemented in C++ language) is available at the main page of BEACON for the command-line use and in Additional file 2. It requires: C-shell compatible shell, make utility, C++ complier, GNU Tar utilities and R language with the “VennDiagram” package. Further information can be found in Additional file 1: Section 1.

## Additional files

Additional file 1:
**Has three sections: section 1 provides documentation for the usage of both web-interface and command-line of the BEACON tool; section 2 explains how to interpret the output in detail including illustration figures; and section 3 shows detailed results of applying BEACON to four different genomes.** (PDF 1809 kb) Additional file 2:
**Contains the source code of BEACON in C++ for command line use along with makefile, a ReadMe file and the license text.** (TGZ 42 kb)

## Abbreviation

BEACON: Automated tool for *B*acterial g*E*nome *A*nnotation *C*omparis*ON*
AMs: Annotation methods
NCBI: National centre for biotechnology information
EA: Extended annotation
EUA: Extended union annotation
PGAAP: Prokaryotic genome automatic annotation pipeline
AAMG: Automatic annotation of microbial genomes
RAST: Rapid annotations using subsystems technology
IGS: Institute for genome science
IMG/ER: Integrated microbial genome expert review
ACS: Annotation confidence score
*H. utahensis*: *Halorhabdus utahensis*
CDS: Coding DNA Sequence
tRNA: Transfer RNA
rRNA: Ribosomal RNA
*E. coli*: *Escherichia coli*
*C. ruddii DC*: *Candidatus Carsonella ruddii DC*

## References

[1] Stein L (2001). Genome annotation: from sequence to biology. Nat Rev Genet.

[2] Yandell M, Ence D (2012). A beginner's guide to eukaryotic genome annotation. Nat Rev Genet.

[3] Stothard P, Wishart DS (2006). Automated bacterial genome analysis and annotation. Curr Opin Microbiol.

[4] Pruitt KD, Tatusova T, Brown GR, Maglott DR (2012). NCBI Reference Sequences (RefSeq): current status, new features and genome annotation policy. Nucleic Acids Res.

[5] Kozak M (1987). An analysis of 5′-noncoding sequences from 699 vertebrate messenger RNAs. Nucleic Acids Res.

[6] Salzberg SL (2007). Genome re-annotation: a wiki solution?. Genome Biol.

[7] Tatusova T, DiCuccio M, Badretdin A, Chetvernin V, Ciufo S, Li W. Prokaryotic Genome Annotation Pipeline. In: The NCBI Handbook. Bethesda (MD): National Center for Biotechnology Information (US); 2013-. 2013. http://www.ncbi.nlm.nih.gov/books/NBK174280/. Accessed 13 Jan 2015.

[8] Alam I, Antunes A, Kamau AA, Ba Alawi W, Kalkatawi M, Stingl U (2013). INDIGO - INtegrated Data Warehouse of MIcrobial GenOmes with Examples from the Red Sea Extremophiles. PLoS One.

[9] Aziz RK, Bartels D, Best AA, DeJongh M, Disz T, Edwards RA et al. The RAST server: Rapid annotations using subsystems technology. Bmc Genomics. 2008;9. doi:10.1186/1471-2164-9-75 10.1186/1471-2164-9-75 PMC2265698 18261238

[10] Pareja-Tobes P, Manrique M, Pareja-Tobes E, Pareja E, Tobes R (2012). BG7: a new approach for bacterial genome annotation designed for next generation sequencing data. PLoS One.

[11] Seemann T (2014). Prokka: rapid prokaryotic genome annotation. Bioinformatics.

[12] Galens K, Orvis J, Daugherty S, Creasy HH, Angiuoli S, White O (2011). The IGS Standard Operating Procedure for Automated Prokaryotic Annotation. Stand Genomic Sci.

[13] Markowitz VM, Szeto E, Palaniappan K, Grechkin Y, Chu K, Chen IM (2008). The integrated microbial genomes (IMG) system in 2007: data content and analysis tool extensions. Nucleic Acids Res.

[14] Klimke W, O’Donovan C, White O, Brister JR, Clark K, Fedorov B (2011). olving the problem: Genome annotation standards before the data deluge. Standards in genomic sciences.

[15] Yang Y, Gilbert D, Kim S (2010). Annotation confidence score for genome annotation: a genome comparison approach. Bioinformatics.

[16] Kasukawa T, Furuno M, Nikaido I, Bono H, Hume DA, Bult C (2003). Development and evaluation of an automated annotation pipeline and cDNA annotation system. Genome Res.

[17] Bakke P, Carney N, Deloache W, Gearing M, Ingvorsen K, Lotz M (2009). Evaluation of three automated genome annotations for Halorhabdus utahensis. PLoS One.

[18] Liu Z, Ma H, Goryanin I (2013). A semi-automated genome annotation comparison and integration scheme. BMC bioinformatics.

[19] Galperin MY, Koonin EV (2004). ‘Conserved hypothetical’ proteins: prioritization of targets for experimental study. Nucleic Acids Res.

[20] R Core Team. R: A Language and Environment for Statistical Computing. R Foundation for Statistical Computing. 2013. http://www.R-project.org/. Accessed 13 Jan 2015.

[21] Chen H, Boutros PC (2011). VennDiagram: a package for the generation of highly-customizable Venn and Euler diagrams in R. BMC bioinformatics.

[22] Anderson I, Tindall BJ, Pomrenke H, Goker M, Lapidus A, Nolan M (2009). Complete genome sequence of Halorhabdus utahensis type strain (AX-2). Stand Genomic Sci.

[23] Nakabachi A, Ueoka R, Oshima K, Teta R, Mangoni A, Gurgui M (2013). Defensive Bacteriome Symbiont with a Drastically Reduced Genome. Curr Biol.

[24] Friedel M, Nikolajewa S, Suhnel J, Wilhelm T (2009). DiProDB: a database for dinucleotide properties. Nucleic Acids Res.

