# Supplementary material for: BEACON: automated tool for Bacterial GEnome Annotation ComparisON
Source: BMC Genomics. 2015 Aug 18;16(1):616. doi: 10.1186/s12864-015-1826-4 (PMC4539851; doi:10.1186/s12864-015-1826-4)
Supplement: Additional file 1: — Has three sections: section 1 provides documentation for the usage of both web-interface and command-line of the BEACON tool; section 2 explains how to interpret the output in detail including illustration figures; and section 3 shows detailed results of applying BEACON to four different genomes. (PDF 1809 kb) [file 12864_2015_1826_MOESM1_ESM.pdf]

ADDITIONAL FILE 1

BEACON: Automated Tool for Bacterial GEnome Annotation ComparisON

Manal Kalkatawi[1,#], Intikhab Alam[1,#] and Vladimir B. Bajic[1,*]

[1] Computational Bioscience Research Center (CBRC), King Abdullah University of Science and Technology (KAUST), Thuwal 23955-6900, Kingdom of Saudi Arabia

Email addresses: manal.kalkatawi@kaust.edu.sa, intikhab.alam@kaust.edu.sa and vladimir.bajic@kaust.edu.sa
*Corresponding author
#Equal contributions

Table of Contents

1 Tool documentation 2
1.1 Guidelines for web interface usage 2
1.2 Guidelines for command line usage 5
1.2.1. OS and Software Install pre-requisites 5
1.2.2. System Configuration Files 5
1.2.3. ReadMe File 5
2 Output description 7
2.1 Annotations information 7
2.2 Comparison to reference 8
2.3 Extended annotations 10
2.4 Venn Diagram 11
2.5 Web 11
3 Detailed comparison of different annotations for four bacterial genomes 12
3.1 H. utahensis 12
***3.2 E. coli* K-12** 14
***3.3 E. coli* TY2482** 17
3.4 C. ruddii DC 20
Supplementary References 23

1 Tool documentation

BEACON is a tool used to compare different annotations for a single bacterial genome. Such annotations may be generated by multiple annotation methods (AMs). BEACON can generate extended annotations through combination of individual ones.

BEACON is available as a web-based tool and the source code is also available for command line use. Annotations of four genomes generated by multiple AMs in the GenBank format can be downloaded from "http://www.cbrc.kaust.edu.sa/BEACON/" home page and used for testing the tool. In what follows we present detailed guidelines for the usage of both command line and web interface.

1.1 Guidelines for web interface usage

BEACON is freely accessible at: http://www.cbrc.kaust.edu.sa/BEACON/. In order to use the online version, please follow these steps:

- **Step 1**: Fill the web-form with required data (See Figure S1)
  1. If you have a reference annotation please upload a GenBank file in first browse button
  2. Provide multiple GenBank-formatted annotation files through second browse button
  3. Enter a descriptive name for this comparison
  4. Type a similarity offset; the default number is 2
  5. Click the "Submit" button or to clear the input click "Reset" button
- **Step 2**: The first result page (See Figure S2)
  1. Click "here" to move to the visualization result page
  2. List of result files
- **Step 3**: Visualization result page (See Figure S3)
  1. Download the textual/detailed output
  2. Figures that show the relationship between different annotations
  3. Table that shows the statistics for different annotations
  4. Table that shows the comparison to the reference annotation (this table will be shown only if you have a reference annotation)

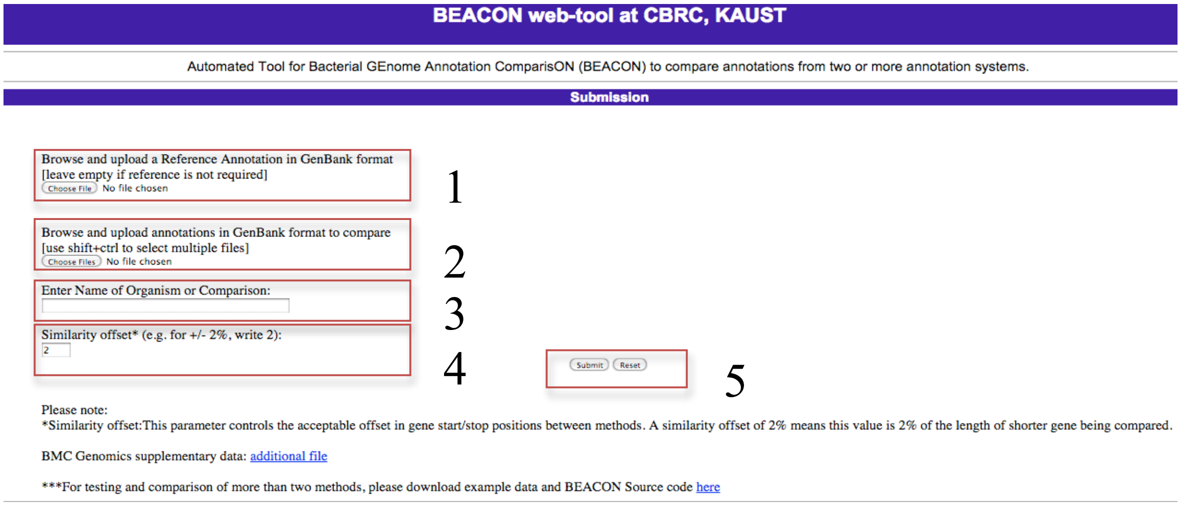

Figure S1: Step 1 of Web interface usage

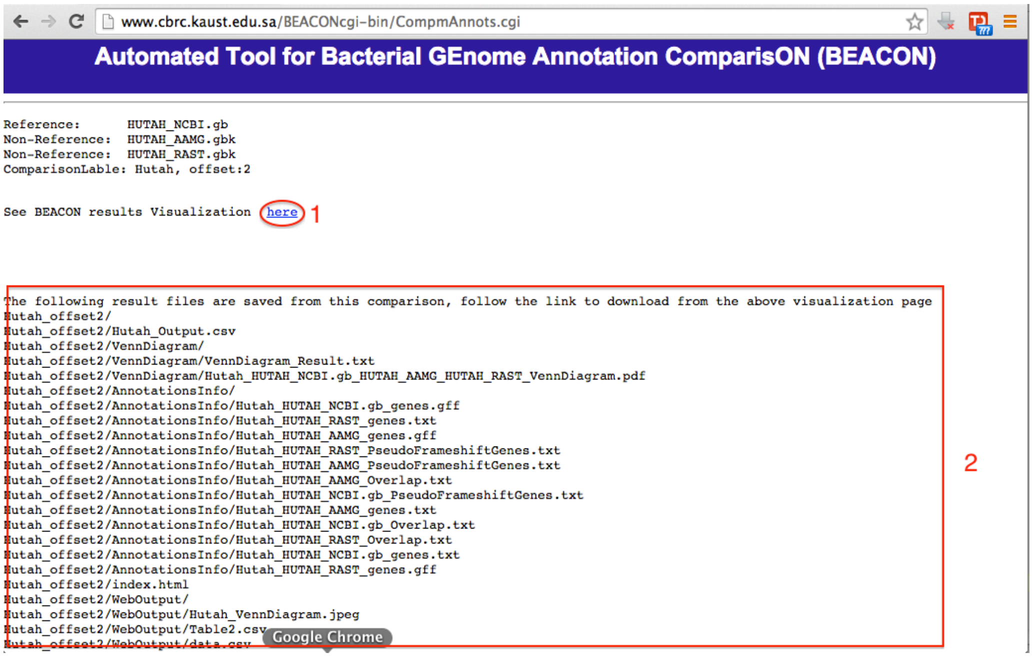

Figure S2: Step 2 of Web interface usage

www.cbrc.kaust.edu.sa/BEACON/AnnotationComp/out/1434754859_Multi_Hutah_offset2/Hutah_offset2/

BEACON webserver at CBRC, KAUST

Download BEACON Annotation Comparison result files archive, here 1

Fig 1. Annotation methods features comparison stats (Histogram)

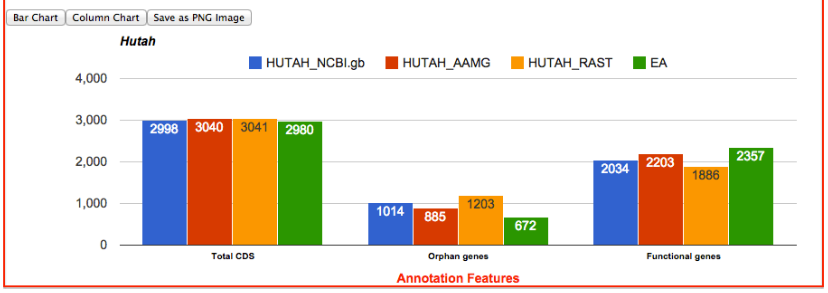

2

Table 1. Annotation methods detailed features comparison

Annotation Features	HUTAH_NCBI.gb Original	HUTAH_NCBI.gb Complemented by other AMs	HUTAH_AAMG Original	HUTAH_AAMG Complemented by other AMs	HUTAH_RAST Original	HUTAH_RAST Complemented by other AMs	EA	Unique	EUA
CDS	2998	2998	3040	3040	3041	3041	2980	698	
rRNA	4	4	3	3	3	3	4	0	
tRNA	45	45	45	45	45	45	45	0	
ncRNA	1	1	0	0	0	0	0	1	
frameshift/Pseudo	0	0	0	0	0	0			
Total	3048	3048	3088	3088	3089	3089	3029	699	
Orphan genes	1014 (33.27%)	777 (25.49%)	885 (28.66%)	837 (27.10%)	1203 (38.94%)	819 (26.51%)	672 (22.19%)	399 (57.08%)	1071 (28
Functional genes	2034 (66.73%)	2271 (74.51%)	2203 (71.34%)	2251 (72.90%)	1886 (61.06%)	2270 (73.49%)	2357 (77.81%)	300 (42.92%)	2657 (71

3

Fig 2. Venn Diagram Showing Unique/Common genes from different Methods

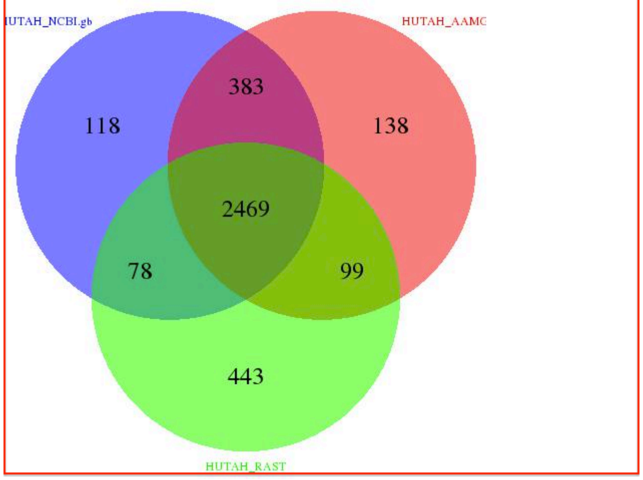

2

Table 2. Comparison of Annotation features to Rerefence 4

Gene calls	Genes annotated by HUTAH_AAMG	% of HUTAH_NCBI.gb genes	Genes annotated by HUTAH_RAST	% of HUTAH_NCBI.gb genes
Detected identical	2780 (CDS= 2732 rRNA= 3 tRAN= 45 ncRAN= 0)	91.21%	2421 (CDS= 2376 rRNA= 0 tRAN= 45 ncRAN= 0)	79.43%
Detected similar	71 (CDS= 71 rRNA= 0 tRAN= 0 ncRAN= 0)	2.33%	126 (CDS= 123 rRNA= 3 tRAN= 0 ncRAN= 0)	4.13%
FN - Short overlap	32	1.05%	74	2.43%
FN - No overlap	165	5.41%	427	14.01%
FP - Short overlap	0		13	
FP - No overlap	237		529	
Similarity score		92.93%		83.00%

Figure S3: Step 3 of Web interface usage

1.2 Guidelines for command line usage

BEACON source code is available for download in the home page of: http://www.cbrc.kaust.edu.sa/BEACON/. In what follows, some instructions to run/use the command line version.

1.2.1. OS and Software Install pre-requisites

Most of the software mentioned here is standard on many UNIX/LINUX systems. To build and use BEACON you will need:

- C-shell compatible shell
- Make utility
- C++ complier
- GNU Tar utilities
- R language with the "VennDiagram" package

1.2.2. System Configuration Files

No special configuration is required.

1.2.3. ReadMe File

```
BEACON: Automated Tool for Bacterial Genome Annotation Comparison. Version 1.1 22/Jan/2015


WHAT IS IT?
-----------

BEACON is a software tool that compares annotations of a particular genome from different
Annotation Methods (AMs).

It uses GenBank format as input and derives Extended Annotation (EA) along side listing
original annotations from individual AMs.


COMMAND LINE VERSION
--------------------

Here we include the source code of BEACON tool written in C++ language.


INSTALLATION
------------

BEAOCN is able to run on any linux platform. To run BEACON to compare annotations from a
genome X with annotations available from AM A and AM B you need to go through the following
steps:

    1. Open a new terminal, download BEACON source and unzip the BEACON_Source.tgz like:
       tar -xzvf BEACON_Source.tgz

    2. Go to the directory that contains BEACON_Source folder. For example:
       cd BEACON_Source/

    3. Use the make Or recompile the source code using the following command:
       g++ BEACON.cpp -o BEACON
```

4. Running BEACON, command line options:

```
./ BEACON <outdirName> <genome label> <offset percentage> [-r] <GenBank file A> <label for Annotation system A> <GenBank file B> <label for Annotation system B> ...
```

Description of the 8 options in BEACON (order is important) mentioned in step 4 above:

1. Path where do you want to save the output and the result files
2. Short label or descriptive name of the genome
3. Offset percentage for the overlap for including overlapping gene based annotations in extended annotation derivation
4. An option [-r] just in case you have a reference
5. Full path to the GenBank file A
6. Label or short descriptive name for annotation method A
7. Full path to the GenBank file B
8. Short descriptive name for annotation method B

NOTE: if you choose to have a reference, you need to insert -r option and the first input annotation will be considered as the reference annotation.

EXAMPLE:

Try BEACON on annotations from different AMs e.g. AAMG and RAST for Halorhabdus utahensis (HUTAH) genome, considering NCBI annotations as reference (required data is included in this package).

```
./BEACON HUTAH_BEACON_OUTPUT/ HUTAH 2 -r BEACON_examples/HUTAH_NCBI.gbk NCBI BEACON_examples/HUTAH_AAMG.gbk AAMG BEACON_examples/HUTAH_RAST.gbk RAST
```

CONTACTS

- If you want to report bugs or have general queries email to <manal.kalkatawi@kaust.edu.sa>
- If you want freely available online version of BEACON please visit: <http://www.cbrc.kaust.edu.sa/BEACON/>

2 Output description

BEACON output is categorized into five groups: ‘annotations information’, ‘comparison to reference’ (only in the case of reference-based comparison), ‘extended annotations (EA, EUA, unique)’, ‘Venn diagram’ and ‘Web’, as shown in Figure S1. The main output folder also contains a comma-separated-value (csv) file that describes the statistical and comparison output.

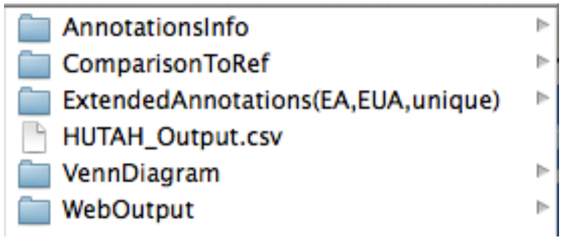

Figure S4: Output folders

2.1 Annotations information

There are four files per annotation method (AM): one for all genes in tabular and detailed format, and the other one for overlapping genes within the annotation of that AM. The naming of the files is (GenomeName_AnnotationName_Information.txt).

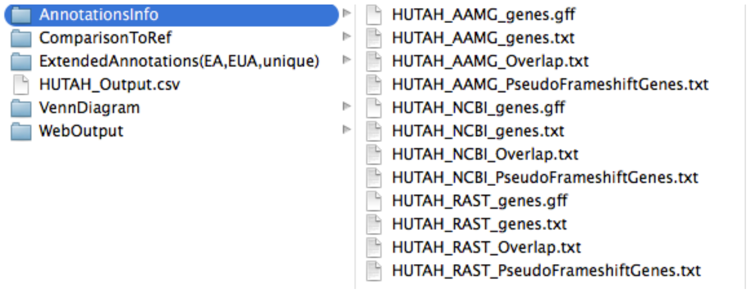

Figure S5: Content of ‘Annotations’ folder

Example of the content of all genes files is shown below, where GeneID is derived as (LocusName:GeneLocation:GeneType).

Gene ID	Locus Tag	Product Name	Type	Start	Stop	Extended annotations	Which AM	Discontiguou	FrameShiftte	Overlapping Status
CP001687:73..2172:CDS	HUTAH_00001	protein cdch	CDS	73	2172			no	no	
CP001687:complement(156529..158010):CDS	HUTAH_00164	hypothetical protein	CDS-hypothetical	156529	158010	predicted d-tagaturonate epimerase	RAST	no	no	
CP001687:109378..110253:CDS	HUTAH_00122	anti-sigma-w factor rsiw protein	CDS	109378	110253			no	no	Short overlap to "HUTAH_00121"

Figure S6: Example of "all genes" file in 'Annotations' folder

Example of the content of overlapping genes files is:

Finding overlaps between genes in AAMG annotation						
**Gene ID**	**Locus Tag**	**Product Name**	**Type**	**Start**	**Stop**	**Overlapping Status**
CP001687:complement(9884..13504):CDS	HUTAH_00012	hypothetical protein	CDS, hypothetical	9884	13504	Short overlap to "HUTAH_00013"
CP001687:complement(13495..14484):CDS	HUTAH_00013	hypothetical protein	CDS, hypothetical	13495	14484	

Figure S7: Example of "overlap" file in 'Annotation' folder

2.2 Comparison to reference

This folder is generated only in the case of a reference-based comparison. The comparison is pair-wise to the reference and the output is files for: unique genes, identical genes, similar genes and all genes.

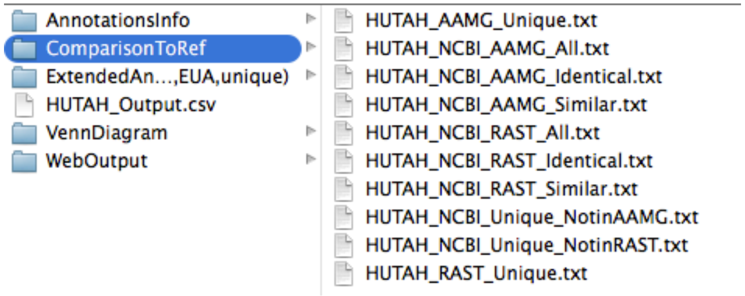

Figure S8: Content of 'Comparison' folder

For 'identical genes' file, the two identical genes are listed where the first one belongs to the reference and the second one belongs to the annotation in the comparison, each with its information.

Those are the identical genes; the first gene belongs to NCBI and the second one belongs to AAMG

Gene ID	Locus Tag	Product Name	Type	Start	Stop	Discontiguous	FrameShiftted	Status
CP001687:73..2172:CDS	Huta_0001	vesicle-fusing atpase	CDS	73	2172	no	no	Identical
CP001687:73..2172:CDS	HUTAH_00001	protein cdch	CDS	73	2172	no	no	Identical

Figure S9: Example of “identical” file in ‘Comparison’ folder

For ‘similar genes’ file, the two similar genes are listed where the first one belongs to the reference and the second one belongs to the annotation in the comparison, each with its information.

Those are the similar genes; the first gene belongs to NCBI and the second one belongs to AAMG

Gene ID	Locus Tag	Product Name	Type	Start	Stop	Discontiguous	FrameShiftted	Status
CP001687:complement(35427..37268):CDS	Huta_0036	molybdenum cofactor synthesis domain protein	CDS	35427	37268	no	no	Similar
CP001687:complement(35427..37277):CDS	HUTAH_00037	molybdopterin molybdenumtransferase protein	CDS	35427	37277	no	no	Similar

Figure S10: Example of “similar” file in ‘Comparison’ folder

For ‘unique genes’ file, all the genes that are found only in that annotation are listed. It is also declared that whether the gene is unique with or without overlap.

Gene ID	Locus Tag	Product Name	Type	Start	Stop	Discontiguous	FrameShiftted	Status
CP001687:3090067..3091347:CDS	Huta_3000	trka-c domain protein	CDS	3090067	3091347	no	no	Unique with overlap
CP001687:complement(3082..3333):CDS	Huta_0004	hypothetical protein	CDS-hypothetical	3082	3333	no	no	Unique_NCBI

Figure S11: Example of “unique” file in ‘Comparison’ folder

For ‘all’ file, all identical and similar genes between the reference annotation and the annotation in the comparison with the addition of the unique genes to each of the annotations are combined.

Gene by gene comparative analysis between NCBI and AAMG annotations for HUTAH genome
The first gene belongs to NCBI and the second one belongs to AAMG

Gene ID	Locus Tag	Product Name	Type	Start	Stop	Discontiguou	FrameShiftte	Overlapping Statu	Status
CP001687:73..2172:CDS	Huta_0001	vesicle-fusing atpase	CDS	73	2172	no	no		Identical
CP001687:73..2172:CDS	HUTAH_00001	protein cdch	CDS	73	2172	no	no		Identical
CP001687:1567089..1568210:CDS	Huta_1592	phosphate abc transporter periplasmic substrate- binding protein	CDS	1567089	1568210	no	no		Similar
CP001687:1567092..1568210:CDS	HUTAH_01685	phosphate-binding protein psts	CDS	1567092	1568210	no	no		Similar
CP001687:complement(3082..3333):CDS	Huta_0004	hypothetical protein	CDS-hypothetical	3082	3333	no	no		Unique_NCBI
CP001687:complement(821840..822076):CDS	HUTAH_00914	hypothetical protein	CDS-hypothetical	821840	822076	no	no		Unique_AAMG

Figure S12: Example of “all” file in ‘Comparison’ folder

2.3 Extended annotations

There are four files in extended annotations: one that contains all common genes across all AMs without those genes that are uniquely belong to each annotation (EA); another file that expand EA by adding uniquely annotated genes from other annotations (EUA); the latter also found in clean format where pseudogenes and frameshifted genes are excluded; and a separate file for unique genes only. Each of these files is available in gff format.

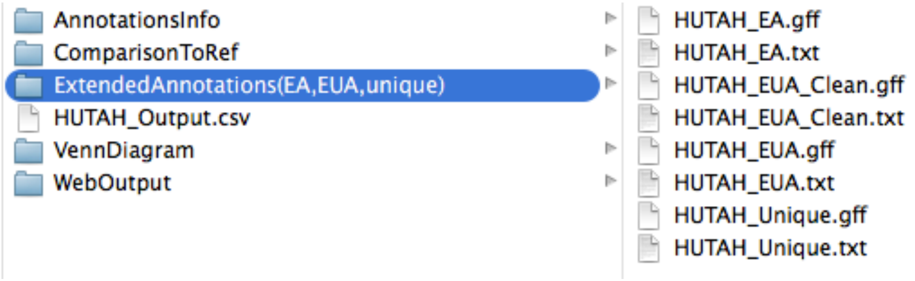

Figure S13: Content of 'Extended annotations' folder

Each of the extended annotations files contains thee pair columns:

(1) *Gene ID* & *Found in*: contains the similar Gene IDs between different AMs separated by (||) sign, followed by the AMs labels for the corresponding gene ids.

(2) *Non-hypothetical annotation* & *which annotation*: contains the functional annotation of this particular gene, followed by the source (AM) of this annotation.

(3) *Hypothetical annotation & which annotation*: contains the hypothetical annotation of this particular gene, followed by the source (AM) of this annotation.

Gene ID	Found in	Non-hypothetical Annotation	Which Annotation	Hypothetical Annotation	Which Annotation
CP001687:73..2172:CDS	NCBI & AAMG & RAST	vesicle-fusing atpase || "protein cdch" || "cell division protein ftsh (ec 3.4.24.-)"	NCBI || AAMG || RAST		
CP001687:1009614..1009686:tRNA	NCBI & AAMG & RAST	trna-gln || "trna-gln" || "trna-gln-ctg"	NCBI || AAMG || RAST		
CP001687:complement(945847..946224):CDS	NCBI & AAMG & RAST	pilt protein domain protein || "pilt protein"	NCBI || AAMG	hypothetical protein	RAST

Figure S14: Example of "EA" file in 'Extended annotations' folder

2.4 Venn Diagram

It contains Venn diagram *per se* along with textual description of the numbers that represent each sector of the Venn diagram.

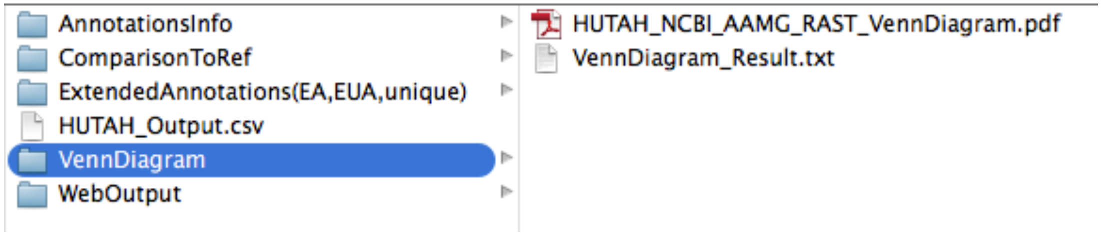

Figure S15: Content of 'VennDiagram' folder

2.5 Web

Google's chart API [1] is used to display the output in bar or column chart along with tables of the statistical and comparison data; the required csv files for generating those charts and tables are found in the Web folder. It also contains the Venn diagram in jpeg format to be displayed in good quality in the webpage.

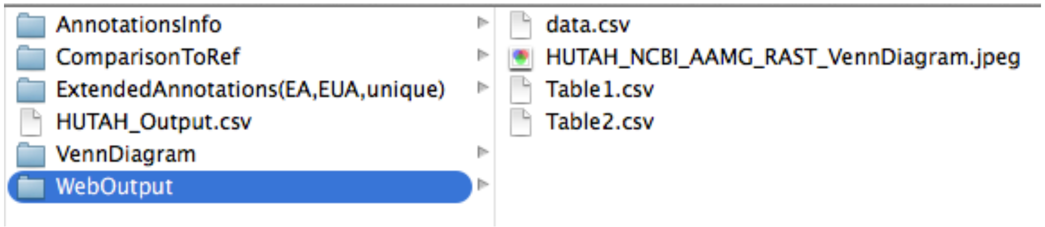

Figure S16: Content of 'Web' folder

3 Detailed comparison of different annotations for four bacterial genomes

To illustrate the capabilities of BEACON, we used three genomes other than the one explained in the manuscript which is *Halorhabdus utahensis (H. utahensis)*. These datasets and their annotations were taken from [2], namely *Escherichia coli (E. coli)* K-12 strain, *E. coli* TY2482 strain and *Candidatus Carsonella ruddii DC* (*C .ruddii DC*). Here we also included RAST [3] as an additional AM and annotated each one of the genomes mentioned in this study through it. The AAMG [2] and RAST annotations were compared against the NCBI [4] annotation for both *E. coli K-12* and *C. ruddii DC*. For *E. coli TY2482*, AAMG, RAST and BG7 [5] annotations were compared against BROAD annotation [6]. The comparison results of these genomes are shown bellow; the similarity offset that was used is 2%.

Note that a very partial comparison of the AAMG annotation of *E. coli* K-12*, E. coli* TY2482 and *C. ruddii DC* with the reference annotations was presented in Table 2 of [2]. Here, however, contrary to [2] we present much more comprehensive comparison of a larger number of annotations (e.g. RAST annotations are included) with results of extended annotations not available in [2].

3.1 H. utahensis

Table S1 Statistics for different annotations for *H. utahensis* genome along with the extended annotations. For orphan and functional genes we show the actual number of genes and the percentage relative to the total number of annotated genes.

Annotation Features	NCBI	AAMG	RAST	Extended Annotations		
				EA	Unique	EUA
CDS	2998	3040	3041	2980	698	3678
rRNA	4	3	3	4	0	4
tRNA	45	45	45	45	0	45
ncRNA	1	0	0	0	1	1
Pseudo/framshift	0	0	0	0	0	0
Total	3048	3088	3089	3029	699	3728
Orphan genes	1014 (33.27%)	885 (28.66%)	1203 (38.94%)	672 (22.19%)	399 (57.08%)	1071 (28.73%)
Functional genes	2034 (66.73%)	2203 (71.34%)	1886 (61.06%)	2357 (77.81%)	300 (42.92%)	2657 (71.27%)
Conserved (non-hypothetical products)	157	19	160			
Functional with gene symbols	1	892	0			
Functional without gene symbols	2033	1311	1886			
Significant overlapping genes	2	0	0			
Short overlapping genes	684	696	660			
Total overlapping genes	686	696	660			
Discontiguous genes	1	0	0			

Table S2 Individual AM extended information for *H. utahensis* genome

**Annotation Features**	**NCBI**		**AAMG**		**RAST**	
	**Original**	**Complemented by annotation of function from AAMG and RAST**	**Original**	**Complemented by annotation of function from NCBI and RAST**	**Original**	**Complemented by annotation of function from NCBI and AAMG**
Orphan genes	1014 (33.27%)	777 (25.49%)	885 (28.66%)	837 (27.10%)	1203 (38.94%)	819 (26.51%)
Functional genes	2034 (66.73%)	2271 (74.51%)	2203 (71.34%)	2251 (72.90%)	1886 (61.06%)	2270 (73.49%)

Table S3 AAMG and RAST annotations compared to NCBI annotation that is taken as the reference for *H. utahensis* genome. False Negatives (FN) are genes that exist in the NCBI annotation but are not predicted by an AM. False Positives (FP) are genes predicted by an AM but not present in the NCBI annotation.

**Gene calls**	**Genes annotated by AAMG**	**% of NCBI genes**	**Genes annotated by RAST**	**% of NCBI genes**
Detected identical	2780 (CDS= 2732 rRNA= 3 tRAN= 45 ncRNA= 0)	91.21%	2421 (CDS= 2376 rRNA= 0 tRAN= 45 ncRNA= 0)	79.43%
Detected similar	71 (CDS= 71 rRNA= 0 tRAN= 0 ncRAN= 0)	2.33%	126 (CDS= 123 rRNA= 3 tRAN= 0 ncRAN= 0)	4.13%
FN – Short overlap	32	1.05%	74	2.43%
FN – No overlap	165	5.41%	427	14.01%
FP – Short overlap	0		13	
FP – No overlap	237		529	
Total reference	3048		3048	
Total Annotation	3088		3089	
Similarity score		92.94%		83.02%

3.2 *E. coli* K-12

Table S4 Statistics for different annotations for *E. coli* K-12 genome along with extended annotations information. For orphan and functional genes we show the actual number of genes and the percentage relative to the total number of annotated genes.

**Annotation Features**	**NCBI**	**AAMG**	**RAST**	**Extended Annotations**		
				**EA**	**Unique**	**EUA**
CDS	4337	4340	4517	4254	1035	5289
rRNA	22	22	22	22	8	30
tRNA	86	82	86	81	92	173
ncRNA	1	0	0	0	1	1
Pseudo/frameshif	111	54	46	62	49	111
Total	4446	4444	4625	4357	1136	5493
Orphan genes	715 (16.08%)	578 (13.01%)	689 (14.90%)	221 (5.07%)	478 (42.08%)	699 (12.73%)
Functional genes	3731 (83.92%)	3866 (86.99%)	3936 (85.10%)	4136 (94.93%)	658 (57.92%)	4794 (87.27%)
Conserved (non-hypothetical products)	95	15	21			
Functional with gene symbols	3731	3227	0			
Functional without gene symbols	0	639	3936			
Significant overlapping genes	0	0	0			
Short overlapping genes	1080	950	950			
Total overlapping genes	1080	950	950			
Discontiguous genes	1	0	0			

Table S5 Individual AM extended information for *E. coli* K-12 genome

**Annotation Features**	**NCBI**		**AAMG**		**RAST**	
	**Original**	**Complemented by annotation of function from AAMG and RAST**	**Original**	**Complemented by annotation of function from NCBI and RAST**	**Original**	**Complemented by annotation of function from NCBI and AAMG**
Orphan genes	715 (16.08%)	261 (5.87%)	578 (13.01%)	301 (6.77%)	689 (14.90%)	512 (11.07%)
Functional genes	3731 (83.92%)	4185 (94.13%)	3866 (86.99%)	4143 (93.23%)	3936 (85.10%)	4113 (88.93%)

Table S6 AAMG and RAST annotations compared to NCBI annotation that is taken as the reference for *E. coli* K-12 genome. False Negatives (FN) are genes that exist in the NCBI annotation but are not predicted by an AM. False Positives (FP) are genes predicted by an AM but not present in the NCBI annotation.

**Gene calls**	**Genes annotated by AAMG**	**% of NCBI genes**	**Genes annotated by RAST**	**% of NCBI genes**
Detected identical	3876 (CDS= 3876 rRNA= 0 tRAN= 0 ncRNA= 0)	87.18%	3624 (CDS= 3609 rRNA= 15 tRAN= 0 ncRNA= 0)	81.51%
Detected similar	120 (CDS= 106 rRNA= 14 tRAN= 0 ncRAN= 0)	2.70%	153 (CDS= 146 rRNA= 7 tRAN= 0 ncRAN= 0)	3.44%
FN – Short overlap	88	1.98%	132	2.97%
FN – No overlap	362	8.14%	537	12.08%
FP – Short overlap	16		36	
FP – No overlap	432		812	
Total reference	4446		4446	
Total Annotation	4444		4625	
Similarity score		89.90%		83.28%

(a)

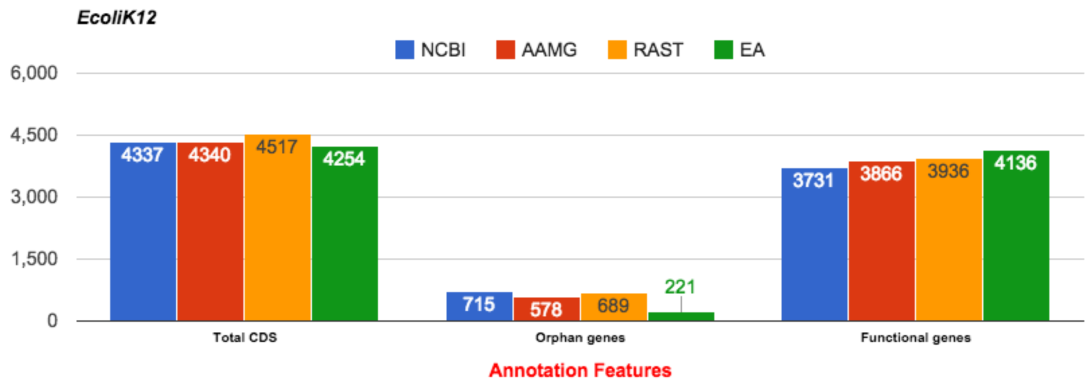

(b)

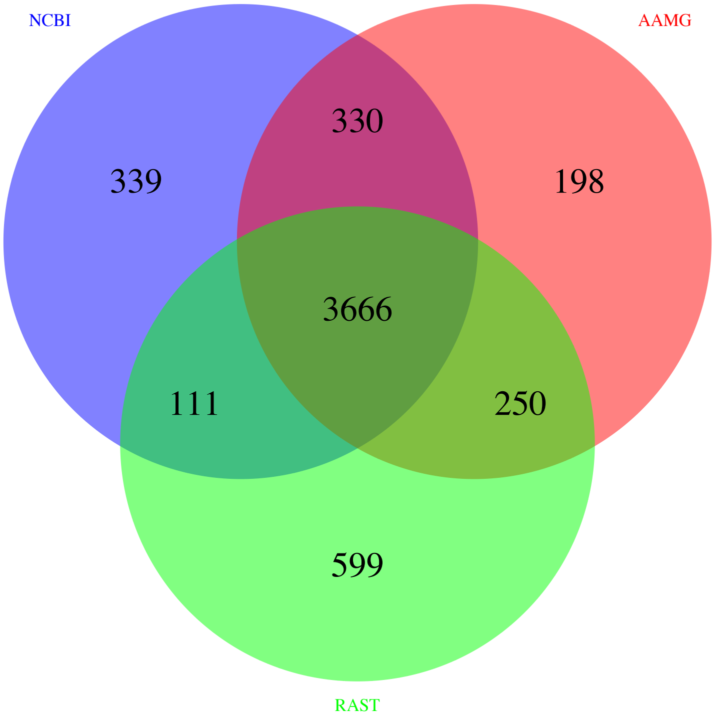

Figure S17: Relationship between NCBI, AAMG and RAST annotations of *E. coli* K-12 genome

3.3 *E. coli* TY2482

Table S7 Statistics for different annotations for *E. coli* TY2482 genome along with extended annotations information. For orphan and functional genes we show the actual number of genes and the percentage relative to the total number of annotated genes.

**Annotation Features**	**BROAD**	**BG7**	**AAMG**	**RAST**	**Extended Annotations**		
					**EA**	**Unique**	**EUA**
CDS	5164	5210	5208	5502	5262	2224	7486
rRNA	22	0	22	22	22	8	30
tRNA	102	0	97	101	96	107	203
ncRNA	0	0	0	0	0	0	0
Pseudo/framshifed	0	1	0	0	0	0	0
Total	5288	5210	5327	5625	5380	2340	7720
Orphan genes	1786 (33.77%)	949 (18.21%)	736 (13.82%)	1082 (19.24%)	341 (6.34%)	1049 (44.83%)	1390 (18.01%)
Functional genes	3502 (66.23%)	4261 (81.79%)	4591 (86.18%)	4543 (80.76%)	5039 (93.66%)	1291 (55.17%)	6330 (81.99%)
Conserved (non-hypothetical products)	0	137	12	42			
Functional with gene symbols	0	0	3125	0			
Functional without gene symbols	3502	4261	1466	4543			
Significant overlapping genes	0	0	0	0			
Short overlapping genes	1648	1212	1658	1678			
Total overlapping genes	1648	1212	1658	1678			
Discontiguous genes	0	0	0	0			

Table S8 Individual AM extended information for *E. coli* TY2482 genome

**Annotation Features**	**BROAD**		**BG7**		**AAMG**		**RAST**	
	**Original**	**Complemented by annotation of function from BG7, AAMG and RAST**	**Original**	**Complemented by annotation of function from BROAD, AAMG and RAST**	**Original**	**Complemented by annotation of function from BROAD, BG7 and RAST**	**Original**	**Complemented by annotation of function from BROAD, BG7 and AAMG**
Orphan genes	1786 (33.77%)	370 (7.00%)	949 (18.21%)	648 (12.44%)	736 (13.82%)	388 (7.28%)	1082 (19.24%)	742 (13.19%)
Functional genes	3502 (66.23%)	4918 (93.00%)	4261 (81.79%)	4562 (87.56%)	4591 (86.18%)	4939 (92.72%)	4543 (80.76%)	4883 (86.81%)

Table S9 BG7, AAMG and RAST annotations compared to BROAD annotation that is taken as the reference for *E. coli* TY2482 genome. False Negatives (FN) are genes that exist in the NCBI annotation but are not predicted by an AM. False Positives (FP) are genes predicted by an AM but not present in the NCBI annotation.

**Gene calls**	**Genes annotated by BG7**	**% of BROAD genes**	**Genes annotated by AAMG**	**% of BROAD genes**	**Genes annotated by RAST**	**% of BROAD genes**
Detected identical	1 (CDS= 1 rRNA= 0 tRAN= 0 ncRNA= 0)	0.02%	5172 (CDS= 5149 rRNA= 22 tRAN= 1 ncRNA= 0)	97.81%	4404 (CDS= 4403 rRNA= 0 tRAN= 1 ncRNA= 0)	83.28%
Detected similar	3802 (CDS= 3802 rRNA= 0 tRAN= 0 ncRAN= 0)	71.90%	2 (CDS= 2 rRNA= 0 tRAN= 0 ncRAN= 0)	0.04%	143 (CDS= 129 rRNA= 14 tRAN= 0 ncRAN= 0)	2.70%
FN – Short overlap	311	5.88%	2	0.04%	131	2.48%
FN – No overlap	1174	22.20%	112	2.12%	610	11.54%
FP – Short overlap	184		0		45	
FP – No overlap	1223		153		1033	
Total reference	5288		5288		5288	
Total Annotation	5210		5327		5625	
Similarity score		72.45%		97.48%		83.33%

(a)

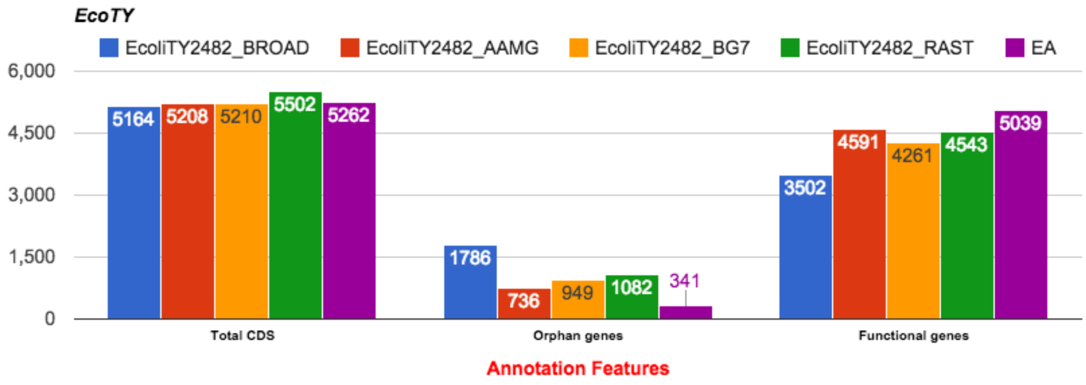

(b)

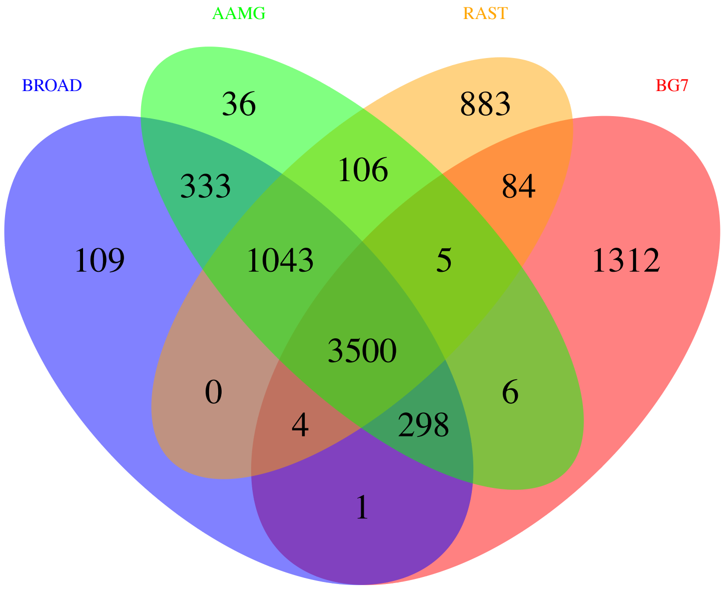

Figure S18: Relationship between BROAD, BG7, AAMG and RAST annotations of *E. coli* TY2482 genome

3.4 C. ruddii DC

Table S10 Statistics for different annotations for *C ruddii DC* genome along with extended annotations information. For orphan and functional genes we show the actual number of genes and the percentage relative to the total number of annotated genes.

Annotation Features	NCBI	AAMG	RAST	Extended Annotations		
				EA	Unique	EUA
CDS	207	190	203	200	36	236
rRNA	3	3	2	3	1	4
tRNA	28	27	27	27	2	29
ncRNA	0	0	0	0	0	0
Pseudo/framshif	0	0	0	0	0	0
Total	238	220	232	230	39	269
Orphan genes	47 (19.75%)	38 (17.27%)	74 (31.90%)	31 (13.48%)	22 (56.41%)	53 (19.70%)
Functional genes	191 (80.25%)	182 (82.73%)	158 (68.10%)	199 (86.52%)	17 (43.59%)	216 (80.30%)
Conserved (non-hypothetical products)	0	0	0			
Functional with gene symbols	128	88	0			
Functional without gene symbols	63	94	158			
Significant overlapping genes	0	0	0			
Short overlapping genes	262	226	230			
Total overlapping genes	262	226	230			
Discontiguous genes	0	0	0			

Table S11 Individual AM extended information for *C. ruddii DC* genome

Annotation Features	NCBI		AAMG		RAST	
	Original	Complemented by annotation of function from AAMG and RAST	Original	Complemented by annotation of function from NCBI and RAST	Original	Complemented by annotation of function from NCBI and AAMG
Orphan genes	47 (19.75%)	34 (14.29%)	38 (17.27%)	31 (14.09%)	74 (31.90%)	40 (17.24%)
Functional genes	191 (80.25%)	204 (85.71%)	182 (82.73%)	189 (85.91%)	158 (68.10%)	192 (82.76%)

Table S12 AAMG and RAST annotations compared to NCBI annotation that is taken as the reference for *C. ruddii DC* genome. False Negatives (FN) are genes that exist in the NCBI annotation but are not predicted by an AM. False Positives (FP) are genes predicted by an AM but not present in the NCBI annotation.

**Gene calls**	**Genes annotated by AAMG**	**% of NCBI genes**	**Genes annotated by RAST**	**% of NCBI genes**
Detected identical	205 (CDS= 177 rRNA= 2 tRAN= 26 ncRNA= 0)	86.13%	206 (CDS= 180 rRNA= 0 tRAN= 26 ncRNA= 0)	86.55%
Detected similar	2 (CDS= 2 rRNA= 0 tRAN= 0 ncRAN= 0)	0.84%	4 (CDS= 2 rRNA= 2 tRAN= 0 ncRAN= 0)	1.68%
FN – Short overlap	22	9.24%	20	8.40%
FN – No overlap	9	3.78%	8	3.36%
FP – Short overlap	2		0	
FP – No overlap	11		22	
Total reference	238		238	
Total Annotation	220		232	
Similarity score		90.39%		89.36%

(a)

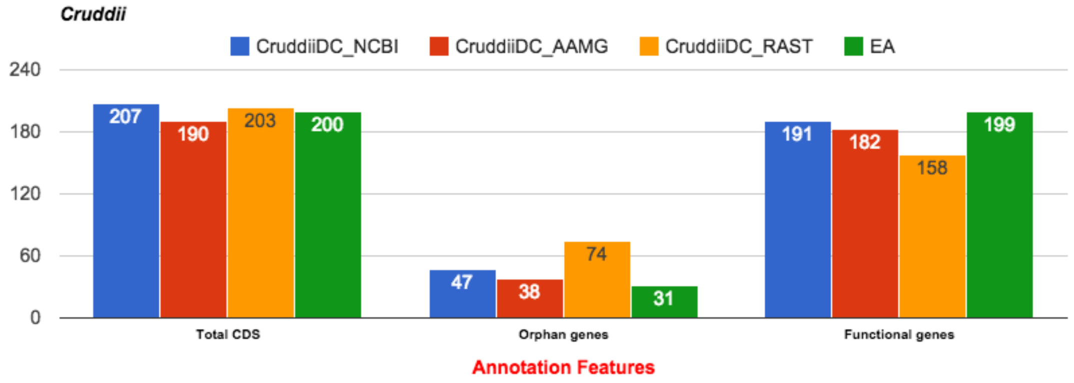

(b)

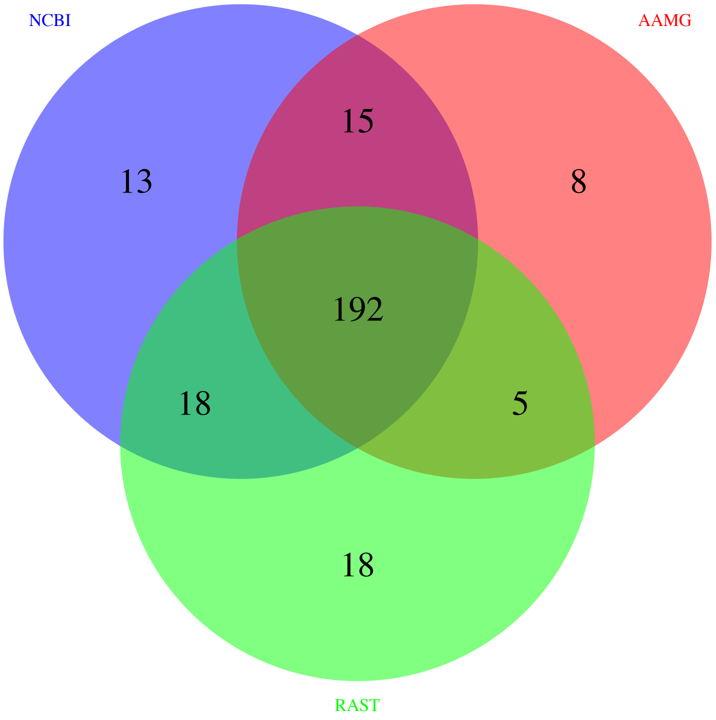

Figure S19: Relationship between NCBI, AAMG and RAST annotations of *C. ruddii DC* genome

Supplementary References

1. Google Charts. https://developers.google.com/chart/. Accessed 13 Jan 2015.

2. Alam I, Antunes A, Kamau AA, Ba Alawi W, Kalkatawi M, Stingl U et al. INDIGO - INtegrated Data Warehouse of MIcrobial GenOmes with Examples from the Red Sea Extremophiles. PloS one. 2013;8(12):e82210. doi:10.1371/journal.pone.0082210.

3. Aziz RK, Bartels D, Best AA, DeJongh M, Disz T, Edwards RA et al. The RAST server: Rapid annotations using subsystems technology. Bmc Genomics. 2008;9. doi:10.1186/1471-2164-9-75.

4. Pruitt KD, Tatusova T, Brown GR, Maglott DR. NCBI Reference Sequences (RefSeq): current status, new features and genome annotation policy. Nucleic acids research. 2012;40(Database issue):D130-5. doi:10.1093/nar/gkr1079.

5. Pareja-Tobes P, Manrique M, Pareja-Tobes E, Pareja E, Tobes R. BG7: a new approach for bacterial genome annotation designed for next generation sequencing data. PloS one. 2012;7(11):e49239. doi:10.1371/journal.pone.0049239.

6. BROAD Institute. http://www.broadinstitute.org/. Accessed 12 Jan 2015.
